# Impact of motivational interviewing on psychosocial and symptom outcomes during breast cancer endocrine therapy

**DOI:** 10.1007/s00520-025-09760-8

**Published:** 2025-08-23

**Authors:** Charlotte Ellis, Katherine Reeder-Hayes, Sarah Drier, Austin R. Waters, Jennifer C. Spencer, Stephanie Wheeler

**Affiliations:** 1 Department of Health Policy and Management, UNC Gillings School of Global Public Health, Chapel Hill, NC USA; 2 University of North Carolina Lineberger Comprehensive Cancer Center, Chapel Hill, NC USA; 3 Division of Oncology, University of North Carolina-Chapel Hill School of Medicine, Chapel Hill, NC USA; 4 Department of Population Health, Dell Medical School, University of Texas at Austin, Austin, TX USA

**Keywords:** Adjuvant endocrine therapy, Breast cancer, Motivational interviewing, Recurrence worry, Psychosocial symptoms

## Abstract

**Purpose:**

Adjuvant endocrine therapy (ET) is recommended to women with hormone receptor-positive (HR +) breast cancer for 5–10 years to reduce recurrence risk and mortality, but adhering to ET for this full period is challenging due to the physical and psychosocial effects of treatment. We sought to understand how participation in a patient-centered counseling intervention affected anxiety and depression, recurrence worry, and treatment related symptoms.

**Methods:**

We conducted a single-arm pilot study over a 12-month period, consisting of five counselor-led motivational interviewing (MI) counseling sessions. Eligible participants were over 18 years old, English speaking, and had stage I-III HR + breast cancer. Survey data collected at baseline and 12 months assessed anxiety and depression, and cancer recurrence worry. Endocrine symptoms were assessed at the 12-month time point, reported descriptively, and age-stratified to examine if symptom burden and age were related.

**Results:**

Of the 42 women who initiated the intervention, 35 completed the baseline and 12-month surveys. Most participants were over 50 (63%), non-Black and non-Hispanic (63%; 97%). Overall patient-reported anxiety and depression decreased from baseline to 12 months, though only the change in anxiety was statistically significant, while cancer worry increased slightly. The most reported endocrine symptoms were hot flashes, night sweats, vaginal dryness, disinterest in sex, and joint pain; endocrine symptoms did not vary by age.

**Conclusion:**

This study shows early promise in the efficacy of MI to improve anxiety, depression, recurrence worry, and treatment-related endocrine symptoms.

## Introduction

Adjuvant endocrine therapy (ET) is typically recommended for women with hormone receptor-positive (HR +) breast cancer [1]. Evidence suggests that taking ET as prescribed for up to 10 years substantially reduces the risk of recurrence and breast cancer-specific mortality [2, 3]. However, many patients struggle to take ET as directed due to the negative physical, psychosocial, and health-related quality-of-life (HRQoL) effects associated with therapy [4, 5]. As a result of these negative effects, ET discontinuation or non-adherence is common and attenuates its potential benefits for women [4, 6–10]. Interventions that address patient barriers are needed to support women undergoing adjuvant ET.

Motivational interviewing (MI) is a patient-centered, focused, and goal-directed counselling approach that aims to define a patient’s motivators and connect them to a behavior to facilitate behavior change [11]. In the context of ET, MI aims to elicit the core value and motivation behind the decision to take ET and help participants to connect future decisions back to that core value or motivator [12]. Behavioral theory explains that MI helps participants build the skills necessary to keep them on track with ET even when barriers arise [12, 13]. MI as a component of behavioral interventions has been shown to improve outcomes across many behaviors and contexts by increasing self-efficacy [14], and has been efficacious in improving medication adherence and quality of life in other diseases. This approach may help in supporting ET use, as increasing self-efficacy may reduce the burden of negative psychosocial and HRQoL effects and motivate continued adherence [12–16].

Inadequately managed ET symptoms and high cancer recurrence worry have been associated with reduced quality of life, greater symptom interference, and ET discontinuation among breast cancer survivors [16–19]. MI may reduce the burden of negative treatment-related anxiety, depression, and cancer worry impacts by encouraging women to talk about their experiences and providing them with external support [12, 14, 17]. Therefore, an MI intervention that improves self-efficacy and communication has the potential to improve overall well-being, in addition to adherence, in women undergoing ET.

MI has been used to address lifestyle behaviors in cancer patients and survivors, but has not been extensively tested in women taking ET [20]. Guiding Endocrine Therapy Success through Empowerment & Technology (GETSET) is an MI-based intervention designed to improve ET taking behaviors in women with HR + breast cancer. This intervention was designed as a flexible approach to help support a diverse array of needs among ET patients [12]. A single-arm study was conducted to preliminarily assess feasibility, acceptability, and potential efficacy of the GETSET intervention. A previous paper on the GETSET pilot study described the early success of MI in improving endocrine therapy adherence [12]. Here, we examine additional potential benefits of the GETSET intervention beyond adherence, by evaluating changes over time and from pre-intervention to post-intervention in patient-reported outcomes including health-related anxiety and depression, cancer recurrence worry, and treatment-related symptoms among participants in the GETSET pilot study.

## Methods

### Study overview

The GETSET study was a single-arm pilot study conducted over a 12-month period investigating the feasibility, acceptability, and efficacy of an MI intervention to increase ET adherence. Participants were recruited from one large academic medical center and four affiliated community clinics within the University of North Carolina’s Cancer Care Network. At the first study visit, participants consented to abstraction of their cancer history data and medical records and completed a baseline survey to provide sociodemographic and self-efficacy data. Participants were sent follow-up surveys electronically or by mail at 6 and 12 months after the baseline visit. Participants received USD 20, USD 30, and USD 50 for completion of the baseline, 6-month, and 12-month survey, respectively. Patients were given an electronic pill cap to monitor their medication adherence.

### Study population

Patients were eligible if they were over 18 years old, could read and speak English, and had stage I-III endocrine receptor-positive breast cancer. Patients were approached by the study team at either the appointment at which they received an ET prescription or within 6 weeks of receiving an ET prescription. Patients could begin the intervention after the completion of all surgery, radiation therapy, and chemotherapy treatments. All participants identified as women.

### Intervention

The GETSET (Guiding Endocrine Therapy Success through Empowerment and Teamwork) intervention program was adapted from previous ET patient qualitative studies and MI-based medication adherence interventions in other disease settings [21–23]. The intervention consisted of five counselor-led sessions, the first of which was delivered in-person at baseline and lasted on average 60–90 min. The subsequent sessions were delivered via telephone at 3, 6, 9, 12, and 24 weeks post-initiation of the intervention, and lasted on average 30–60 min. Participants were assigned to an MI counselor that conducted each session. Counselors were study administrators who were trained in MI during a 2-day workshop. To ensure fidelity to the intervention, counselors used an interview guide to direct participants through sessions with notations for section completion and participated in routine debriefing sessions with an MI supervisor/trainer. Each session was structured to adapt to patient concerns, identify ET knowledge and motivations, and improve self-efficacy. Participants were guided through activities that encouraged them to set goals and identify support systems and ways to overcome the challenges of treatment. A workbook was provided to patients to guide them through the activities and content of MI sessions. The workbooks also had online and local resources for breast cancer survivors.

### Data collection

The baseline and 12-month surveys collected data on participants’ anxiety, depression, and recurrence worry. The Patient-Reported Outcomes Measurement Information System (PROMIS) scale was used to measure general HRQoL related to anxiety and depression at each time point [24–26]. The PROMIS Short Form v1.0–Anxiety 6a (PROMIS-A) and Depression 6a (PROMIS-D) were administered to participants [25, 26]. Participants reported whether PROMIS statements applied to them on a 5-point Likert scale of “never” to “always” in the previous 7 days. Participants also completed the Cancer Worry Scale (CWS) to report their feelings about cancer recurrence worry on a 4-point scale of “never,” “sometimes,” “often,” or “always.” CWS scores range from 8 to 32, with higher scores indicating greater cancer worry [19].

At the 12-month time point, participants reported their endocrine-related symptoms. This instrument was measured with agreement to statements about symptoms over the past 7 days on a 5-point Likert scale ranging from “not at all” to “very much.” Aggregated endocrine symptom scores ranged from 0–76, with higher scores indicating higher symptom burden.

### Outcomes and analysis

The primary outcome of this analysis was changes in responses to anxiety, depression, and cancer worry constructs from baseline to 1 year. Responses to each instrument were converted to a numerical label and aggregated. T-scores for both PROMIS measures were determined using the HealthMeasures Scoring Service and averaged. The T-score metric utilizes 50 as the mean of the relevant reference population and 10 as the standard deviation. Higher T-scores indicate more of a concept being measured. A T-score within normal limits is less than 0.5 standard deviations from the mean. CWS scores were determined for each participant and averaged across the sample. A two-way *t*-test was conducted to analyze the difference in means for responses to the instruments. An alpha level of 0.05 was used to determine statistical significance of mean differences.

A secondary outcome was to explore how the reported burden of endocrine symptoms, measured by FACT-ES score, differed by age. Responses to this instrument were dichotomized as little to no endocrine symptom burden versus moderate to severe symptom burden, to reflect the fact that moderate to severe symptoms are associated with greater morbidity, whereas “little to no” symptoms have clinically little impact on survivors’ wellbeing. These data were stratified by participants over the age of 50 compared to under the age of 50 since age-related menopause is an important determinant of type of ET prescribed, and age is an important correlate of non-ET related menopausal symptoms that are similar to those experienced with ET. Participants who were exactly 50 were included in the over 50 age group. A Fisher’s exact test was conducted at an alpha level of 0.05 to see if age and symptom burden were related. All study instruments are validated, reliable scales [25–27]. All analysis was done in R version 4.3.1.

### IRB statement

Study activities were approved by the University of North Carolina’s Institutional Review Board (IRB #13–0736) and informed consent was obtained.

## Results

Forty-six women consented to the GETSET study. Of these, four women withdrew or were lost to follow-up prior to initiating the intervention. Of the remaining 42 who initiated the intervention, four additional women withdrew and three were lost to follow-up. A total of 35 women completed the baseline survey and 35 completed the 12-month survey, with 32 women completing both surveys.

Participant characteristics are outlined in Table 1. At baseline, 13 (37%) participants were under the age of 50, and 22 (63%) were over the age of 50. The majority of participants were non-Black (63%) and non-Hispanic (97%). The most common relationship status was married or living with a partner (69%), though this was more common in the younger age group (84% of under 50; 59% of over 50). Most women had at least a 4-year college degree (74%), with a slightly greater percentage of those under 50 (85%) having a 4-year degree or higher compared to those over 50 (68%). Fourteen (40%) participants reported an annual household income greater than 100,000 USD. Finally, most participants were privately insured (92% under 50; 64% over 50).

**Table 1:** GETSET participant characteristics by age

	Total (*N* = 35)	< 50 years (*n* = 13)	≥ 50 years (*n* = 22)
	***N* (%)**	***N* (%)**	***N* (%)**
**Race**			
Black	13 (37)	4 (31)	9 (41)
Non-Black	22 (63)	9 (69)	13 (59)
**Ethnicity**			
Non-Hispanic	34 (97)	13 (100)	21 (95)
**Marital status**			
Not living with a partner	11 (31)	2 (16)	9 (41)
Married, or living with a partner	24 (69)	11 (84)	13 (59)
**Any dependents in household**	18 (51)	10 (77)	8 (36)
**Educational attainment**			
High school graduate (or GED)	3 (9)	0 (0)	3 (14)
Technical school/some 4-year college	6 (17)	2 (15)	4 (18)
4-year college graduate	18 (51)	8 (62)	10 (45)
Post-graduate/professional degree	8 (23)	3 (23)	5 (23)
**Annual household income**			
Less than $49,999	8 (23)	3 (23)	5 (23)
$50,000 to $99,999	8 (23)	4 (31)	4 (18)
$100,000 +	14 (40)	5 (38)	9 (41)
I prefer not to answer	5 (14)	1 (8)	4 (18)
**Insurance type**			
Uninsured	2 (6)	0 (0)	2 (9)
Private	26 (74)	12 (92)	14 (64)
Public insurance	7 (20)	1 (8)	6 (27)

Non-Black includes White and Other

Table 2 shares the changes to PROMIS and CWS scores during the study period. At baseline, the average PROMIS-Anxiety T-score was 51.1. Over the 12-month period, the average T-score decreased statistically significantly by 4 points to 47.5 (*p*-value = 0.04). The average PROMIS-Depression T-score decreased by 2 points, from 47.8 to 45.8 (*p*-value = 0.1). Average CWS scores increased slightly by 0.7 points, from 11.2 to 11.8 (*p*-value = 0.1). These values exceeded minimal clinically important difference established for all three scales [27].

**Table 2:** PROMIS T-scores and cancer worry scale scores from baseline to 12-month follow-up

	Baseline	12 Months	Change	***p***-value
PROMIS-A	51.16	47.53	− 3.63	0.04*
PROMIS-D	47.81	45.80	− 2.01	0.10
CWS	11.22	11.88	0.66	0.14

*CWS*, Cancer worry scale

*p-value < 0.05

Age-stratified changes in average scores for PROMIS and CWS are shown in Table 3. PROMIS-A T-scores for women under 50 decreased from 53.4 to 49.5. For women over 50, T-scores decreased from 49.9 to 46.4. PROMIS-D T-scores lowered from 49.70 to 47.23 in women under 50, and from 46.83 to 45.06 in women over 50. Women under 50 had higher CWS scores on average, which increased from 13.0 to 14.1 during the study period. CWS scores for women over 50 increased from 10.29 to 10.72. Although none of the stratified findings was statistically significant, possibly due to small sample sizes, the magnitude of PROMIS-A and PROMIS-D scale changes in sub-groups was similar to the magnitude observed in the overall cohort and exceeded established meaningful change thresholds for cancer patients [27].

**Table 3:** Age-stratified PROMIS and Cancer worry scale score changes

	Under 50 ***N*** = 11		Over 50 ***N*** = 21	
	Change	***p*-**value	Change	***p***-value
PROMIS-A	− 3.94	0.2045	− 3.47	0.1195
PROMIS-D	− 2.47	0.3094	− 1.76	0.2259
CWS	1.09	0.2529	0.43	0.3835

Most endocrine symptoms were similar across the two age groups, as shown in Table 4. The most commonly reported symptoms were hot flashes (54%), night sweats (43%), vaginal dryness (46%), disinterest in sex (49%), and joint pain (46%). The average endocrine symptom score for the entire sample was 17.31 (11.59). Women under 50 had slightly numerically greater endocrine symptoms than women over 50, with respective scores of 21.54 and 14.82, but these differences were not statistically significant.

**Table 4:** Symptom frequency among those reporting moderate to severe burden

Endocrine symptom statement	Total (%) *N* = 35	< 50 (%) *N* = 13	≥ 50 (%) *N* = 22	*p*-value
I have hot flashes	19 (54)	8 (62)	11 (50)	0.73
I have lost interest in sex	17 (49)	8 (62)	9 (41)	0.49
I have pain in my joints	16 (46)	7 (54)	9 (41)	0.73
I have vaginal dryness	16 (46)	8 (62)	8 (36)	0.18
I have night sweats	15 (43)	7 (54)	8 (36)	0.48
I have pain or discomfort with intercourse	11 (31)	6 (46)	5 (23)	0.28
I have gained weight	10 (29)	5 (38)	5 (23)	0.45
I have cold sweats	9 (26)	4 (31)	5 (23)	0.70
I have mood swings	9 (26)	6 (46)	3 (14)	0.05*
I have breast sensitivity/tenderness	8 (23)	3 (23)	5 (23)	1.0
I am irritable	8 (23)	5 (38)	3 (14)	0.21
I have vaginal itching/irritation	0 (0)	0 (0)	0 (0)	-
I have vaginal bleeding or spotting	0 (0)	0 (0)	0 (0)	-
**Mean ES score (SD)**	17.31 (11.59)	21.54 (12.76)	14.82 (10.34)	0.0980

Notes: Fisher’s exact test does not show the absence of symptom burden; certain symptom statement results were suppressed to ensure confidentiality;* ES* endocrine Symptom

*p-value < 0.05

## Discussion

The GETSET pilot study provided an opportunity to examine patterns of patient-reported outcomes of endocrine therapy use, including anxiety, depression, symptoms, and cancer worry. Anxiety decreased from pre- to post-intervention, and these patterns were similar in younger and older women. Non-significant trends toward decreased depression and increased cancer worry were also seen between time points. Hot flashes were the highest reported endocrine symptom; 54% of women reported hot flashes as moderately to severely burdensome. Reported symptoms and symptom burden among the GETSET sample are reflective of the literature on endocrine symptoms in women taking ET [28]. While the present study was not powered to examine differences by subgroup, women under 50 had numerically higher burdens of common psychosocial and physical symptoms of breast cancer and its treatment, including anxiety, depression, cancer worry, and endocrine symptoms. Prior studies have also found higher side effect burdens of ET in younger women, which may reflect difference in symptom management, subjective perceptions of burden, or objectively higher burden [29, 30].

Relative to the general population, GETSET participants reported normal anxiety and depression [31]. Though the overall PROMIS-A T-score was within normal limits at each time point, there was a statistically significant decrease in score from baseline to follow-up. This finding suggests that the GETSET intervention may improve anxiety, even when the burden of anxiety is already low. However, it is also possible that anxiety declined over time regardless of intervention, and this hypothesis will be further tested in an ongoing randomized trial. Overall PROMIS-D scores, as well as age-stratified PROMIS scores, were also within normal range, and the study was not powered to evaluate the significance of the relatively small observed changes. However, the numeric change in these items exceeds established minimal important change thresholds [27]. Based on the literature, minimally important change can range from 2 to 6 T-score points, with cancer-specific meaningful change for PROMIS averaging between 2 and 3 points [27]. Given the presence of meaningful change for overall and age-stratified PROMIS-A responses, the data suggests that MI counseling may have helped reduce anxiety. The PROMIS-D scores for participants under 50 met the meaningful change threshold. These data are hypothesis-generating about the potential influence of MI on health-related anxiety and depression, and warrant additional investigation to determine whether meaningful change would be present in a larger sample. Interestingly, the average CWS score increased slightly between time points, despite more time since diagnosis and the receipt of a supportive cancer intervention. The change for women under 50 was greater, which reflects the literature that younger breast cancer survivors have greater fear of recurrence compared to older survivors [32]. The evolution of cancer worry over time among survivors merits further study.

Women under 50 reported numerically more endocrine symptoms on average. Data suggest that endocrine symptoms may be more prominent and burdensome in premenopausal women [33, 34]. These symptoms may be more unfamiliar in younger women than in postmenopausal women who have comparatively dealt with them for longer. Additionally, endocrine symptoms reported by older women may be due to menopause, given that aromatase inhibitors are more associated with musculoskeletal symptoms [34, 35]. The presence of more burdensome endocrine symptoms in younger women may provide evidence as to why ET nonadherence and discontinuation is greater in this age group [36]. Our study adds to a symptom literature providing strong rationale for routine symptom monitoring and patient-reported outcomes (PROs) to be a more established part of the care delivery process. The use of PROs in addition to clinical indicators could improve overall clinical outcomes, anxiety and depression, and symptom management in this population [37–39]. Additionally, greater symptom burden in premenopausal women should be considered when designing ET adherence interventions, as interventions will be more efficacious in this population if they target younger women’s distinct experiences.

Other interventions tested to improve ET adherence and relieve symptom burden in this population focus on improving patient information and education about breast cancer, benefits and side effects of ET, text messaging reminders, and lifestyle changes to relieve symptoms [40–42]. A similar telehealth intervention to GETSET used coping and cognitive skills and showed a reduction in reported symptom burden and an improvement in mood compared to a control group [43]. Other non-pharmacological interventions for endocrine symptoms have included weight management, cognitive behavioral therapy, and acupuncture to address hot flashes, night sweats, and joint symptoms [44–49]. Pharmacological interventions like lubricants and supplements have shown to be helpful in reducing vaginal dryness and sexual dysfunction [50, 51]. While these interventions are feasible and acceptable among participants, few of these studies report measurable change in HRQoL [40, 41, 50]. GETSET is the first MI-based oral medication adherence intervention piloted in this population.

GETSET was designed such that participants could choose each MI session topic. Relevant topics included recurrence fear, symptom worry, lack of adequate support, and feelings of stress. During sessions, participants identified coping strategies and self-efficacy to overcome identified strategies. The structure and topic selection of MI sessions may have targeted participant’s psychosocial burdens, leading to the decreases in PROMIS scores. It is important to note that participants generally had normal anxiety and depression at baseline, so observed changes were minimal. Perhaps for people with worse initial HRQoL, MI counseling could have a more profound impact on their psychosocial symptoms, though this would need to be observed with a larger sample. The minimal changes may also suggest that not all survivors need help with these psychosocial symptoms, and future investigation should consider more comprehensive ways of analyzing HRQoL data.

This study has several limitations. The sample was limited to women in a single health system who self-selected to participate in this intervention. As a result, the study population is not generalizable to the broader population of ET users. The small sample size also limited our ability to detect differences and interpret non-significant findings. Some participants did not complete post-intervention surveys, likely due to the long time lapse between recruitment and measurement of 12-month anxiety and depression outcomes. Additional work with a larger sample would improve precision of estimates. However, this pilot study was conducted to assess feasibility and acceptability of an MI intervention and generate hypotheses for a larger trial. This study provides preliminary evidence for MI affecting health-related anxiety and depression, and fear of recurrence, which can be better tested with the larger study to elucidate whether observed differences are truly due to chance.

In conclusion, ET is a critical adjuvant therapy in women with hormone receptor-positive breast cancer. ET has a high rate of nonadherence, particularly due to the burdensome psychosocial and physical side effects associated with treatment. Motivational interviewing has shown promise in reducing psychosocial burden, as was demonstrated with our data, which has led to the development of an ongoing national trial to investigate these issues more fully (ClinicalTrials.gov ID NCT04379570) [52]. The GETSET intervention was piloted to improve ET use and better understand the effect of MI on medication adherence. This study reinforces the need for interventions that address the complex barriers to ET use to improve survival and survivorship in this population.

## Data Availability

Data Availability Statement: Informed consent was obtained from all study participants. De-identified data are available from the corresponding author on request.
